# Individualized 3D-printed navigation template-assisted tension band wiring for olecranon fractures

**DOI:** 10.1186/s13018-023-03892-5

**Published:** 2023-06-05

**Authors:** Xu Xiong, Ya-Ling Chen, Lan Zhao, Hao Li, Min Xu, Feng Shuang

**Affiliations:** 1 Department of Orthopedic Surgery, The 908th Hospital of the Chinese People’s Liberation Army Joint Logistics Support Forces, No.1028 Jinggangshan Avenue, Qingyunpu District, Nanchang, 330006 Jiangxi Province People’s Republic of China; 2 The First Clinical Medical College of Nanchang University, Nanchang, 330006 People’s Republic of China; 3 Department of Neurology and Orthopedic Intensive Care Unit, The First Affiliated Hospital of Nanchang University, Nanchang, 330006 People’s Republic of China

**Keywords:** 3D-printed navigation template, Tension band wiring, Olecranon fracture, K-wire

## Abstract

**Purpose:**

3D printing techniques guide precision medicine and show great development potential in clinical applications. The purpose of this study was to compare the clinical outcomes of 3D-printed navigation templates versus free-hand in tension band wiring (TBW) procedures for olecranon fractures.

**Methods:**

Patients who underwent TBW due to Mayo type II olecranon fractures between January 2019 and December 2021 in our hospital were prospectively enrolled in the study. The patients were divided into the 3D printed navigation template guiding TBW group (3D printed group) and the free-hand TBW group (free-hand group). The primary endpoint of this study was the success rate of the bicortical placement of Kirschner wires (K-wires). Times of intraoperative fluoroscopy, operation times, complications, VAS scores, and Mayo Elbow Performance Scores (MEPS) were analyzed as the secondary outcomes measure.

**Results:**

The success rate of the bicortical placement of K-wires was 85.7% in the 3D Printed group was significantly higher than the free-hand group (60%). There were fewer times of intraoperative fluoroscopy in the 3D Printed group (1.43 ± 0.51) than that in the free-hand group (2.60 ± 1.00) with statistical significance (*P* < 0.05). At the date of the last follow-up, four patients suffer from pain and skin injury at the K-wires insertion site in the 3D Printed group and 14 patients in the free-hand group, a significant difference between the two groups (*P* < 0.05). No statistically significant differences were found in operation time, VAS scores, and MEPS between the two groups.

**Conclusions:**

The individualized 3D-printed navigation template-assisted TBW demonstrated good accuracy and resulted in reduced times of intraoperative fluoroscopy and complication compared to the free-hand TBW for olecranon fractures.

## Introduction

Olecranon fractures have been estimated to account for 18% of proximal forearm fractures. Mayo type-2 fractures were the most common type of olecranon fractures, accounting for 47% of the fractures [1]. Usually, this type of olecranon fracture is displaced due to contraction and distraction of the triceps tendon. Conservative treatment can be offered in the treatment of low-demand elderly patients, but open reduction and internal fixation are generally considered the best option for olecranon fractures. The aim of surgical treatment includes the reduction of the fracture, and restoring stability to the extensor mechanisms and elbow joint to allow early functional exercise. Various surgical options are available, such as tension band wiring (TBW), plate-and-screw fixation, and intramedullary nail fixation [2]. The TBW is still one of the most common surgical methods because of its low cost and simple operation.

The TBW technique involves parallel insertion of two Kirschner wires (K-wires) parallel inserted from the proximal fracture site of the olecranon and a tension-band wire loop. The distal end of the K-wire was allowed to be placed in the medullary cavity. Although the favorable effects of tension-band fixation for most patients with olecranon fractures, a significant fraction of patients suffer from pain and skin injury at the K-wires insertion site [3]. The main cause of local pain was the displacement of K-wires; K-wires positioned in the medullary cavity were associated with a higher probability of loosening and proximal migration [4]. It is controversial to choose the intramedullary or bicortical placement of K-wires for TBW [5], the bicortical technique of K-wires across the proximal fracture site of the olecranon and penetrated the distal anterior cortex may be a more appropriate choice [6]. Without an accurate auxiliary tool in tension band surgery, the K-wires were inserted based on the experience of physicians. 3D-printed techniques guide precision medicine and show great development potential in clinical applications. Surgery can be performed with models constructed with 3D-printed technology because it enables the rapid construction of accurate, complete fracture models. With the development of this technology and 3D-printed navigation templates have been proposed as alternatives to assisted conventional surgery [7, 8].To our knowledge, up to date, no studies have applied 3D-printed and navigation templates to assist TBW in olecranon fractures.

This investigation aimed to compare the clinical outcomes of a 3D printed navigation template versus free-hand in TBW procedure for olecranon fractures.

## Materials and methods

### Patients

This study is a prospective, non-blinded, controlled, single-centre trial. Patients who underwent TBW due to olecranon fractures between January 2019 and December 2021 in our hospital were enrolled. The inclusion criteria of this study included: (1) patients diagnosed with olecranon fractures (Mayo type 2); (2) aged ≥ 18 years; (3) elbow trauma. The exclusion criteria are as follows: (1) open trauma; (2) pathological fracture; (3) patients with co-morbidities or significant systemic diseases. A total of 51 patients included in this study and were classified into two groups consisting of 21 patients who underwent individualized 3D-Printed template assisted TBW group (3D printed group) and 30 who underwent free-hand TBW surgery group (free-hand group). This study was approved by the Institutional Review Board (IRB) of the Human Research Ethics Committee of our institute (No. 2020LL004).

### Surgical methods

#### 3D-printed template assisted TBW group

A digital picture archive communication system (PACS) at our hospital exported the preoperative CT scans. The Mimics software v10.01 (Materialise, Belgium) was applied to construct the 3D model of olecranon fractures. The 3D model was slowly moved to simulate the intraoperative reduction until a satisfactory reduction. We simulated the K-wire implantation state by placing two parallel 1.6-mm cylinders on the reduction model. To ensure that the cylinder passes through the anterior cortex of the ulna without entering the joint cavity, an individualized guide template assisted was then designed. We printed the olecranon fracture model and guide plate using an optical 3D printer (Jiangxi Xiaoman Technology Co., Ltd.). We performed a simulated operation using a guide plate on a reduced fracture model. The guide plate was sterilized using a low-temperature plasma technique before operation.

**Fig. 1:**
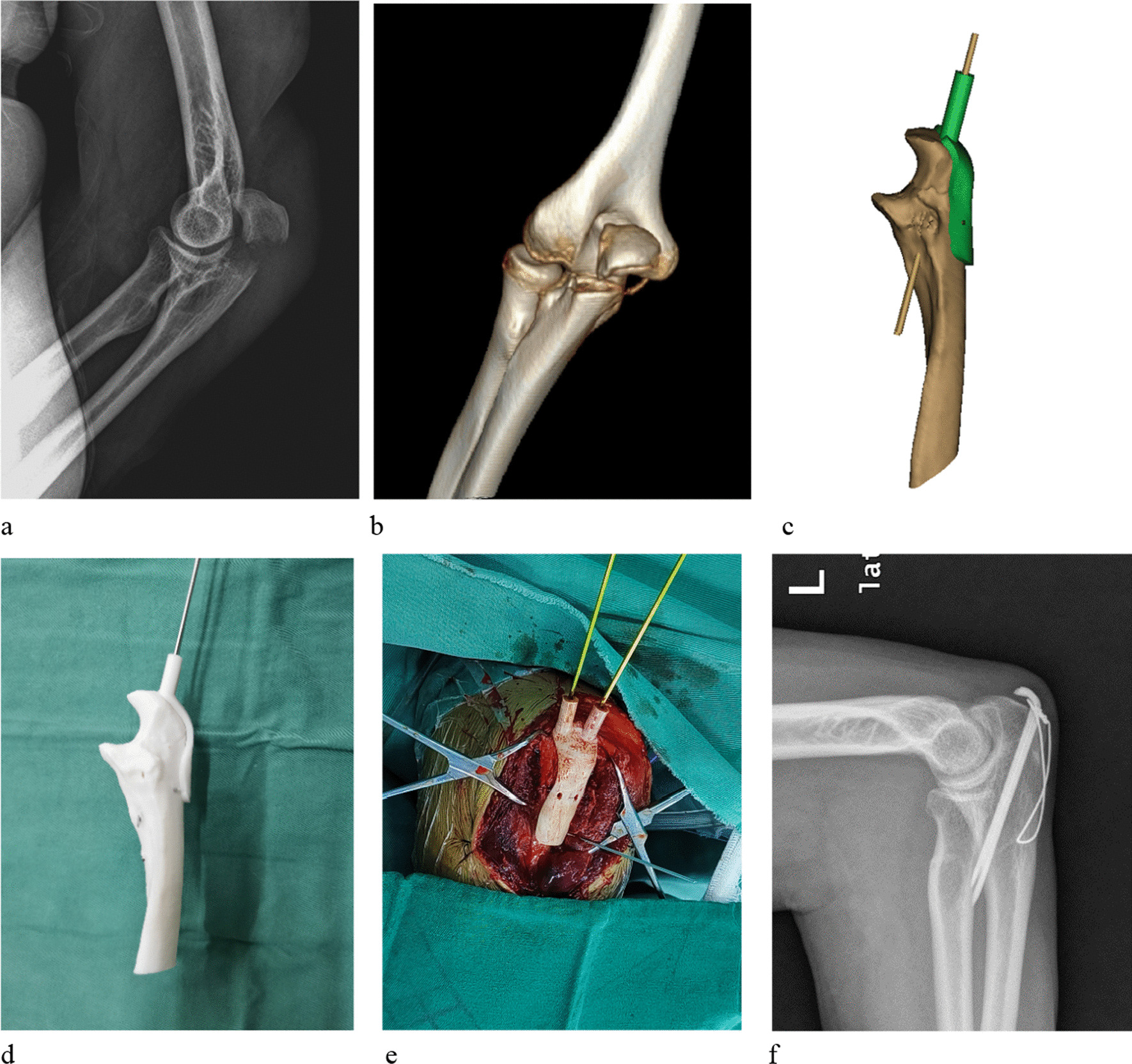
A 49-year-old man treated with individualized 3D-Printed template assisted TBW for olecranon fracture. Lateral-view radiograph (**a**) before surgery. Preoperative 3-dimensional reconstruction image (**b**). Simulated reduction and fixation in software (**c**). Simulated operation with the 3D-printed model and navigation template (**d**). The K-wires were inserted with the assistance of navigation template (**e**). Lateral-view radiograph (**f**) after surgery

After brachial plexus anesthesia or general anesthesia, the skin of the upper limb surgical area was disinfected. A single posterior elbow-curved incision was made to expose the fracture site, and the olecranon fracture was anatomically reduced and initially fixed with two tenaculum clamps. After matching the guide plate to the area of the olecranon, two parallel 1.6 mm K-wires were inserted through the navigation holes. The K-wires were inserted longitudinally across the proximal fracture site of the olecranon and penetrated the distal anterior cortex. A transverse tunnel was drilled into the ulna about 4 cm distal to the fracture line, then a 1.0 or 1.2 mm wire was threaded through the tunnel and around the end of the K-wire to perform a Fig. 8 loop tie. The end of the wire and K-wire were trimmed and inserted into the triceps tendon. The range of motion of the elbow and the strength of internal fixation were examined intraoperatively. The C-arm X-ray was used to examine the fracture reduction quality, the length of K-wire penetration from the anterior cortex, and implantation site. A representative case that underwent 3D-Printed template assisted TBW is shown in Fig. 1.

#### Free-hand TBW group

**Fig. 2:**
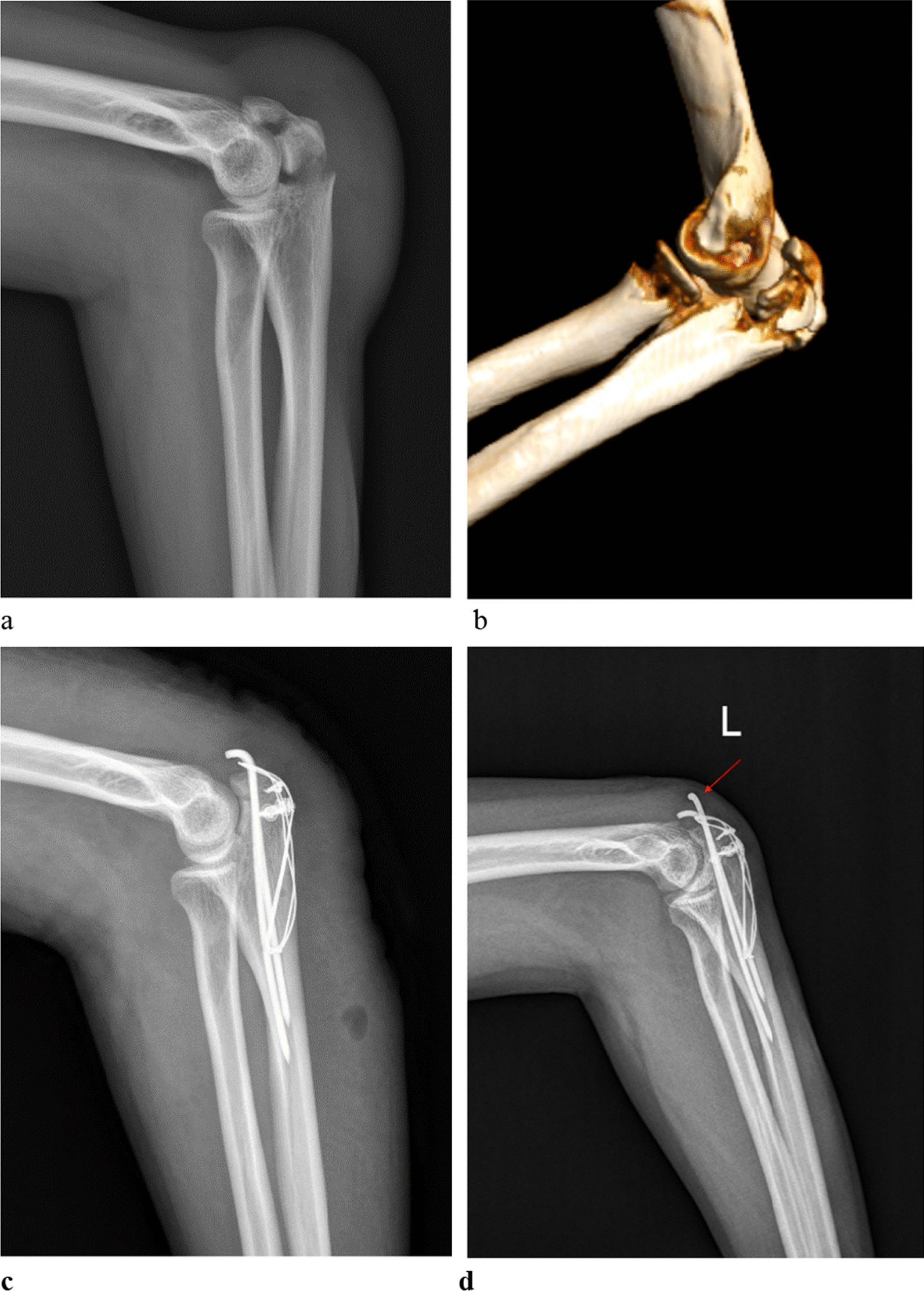
A 33-year-old man treated with free-hand TBW for olecranon fracture. Lateral-view radiograph (**a**) before surgery. Preoperative 3-dimensional reconstruction image (**b**). Immediate post-operative X-ray lateral view (**c**). Lateral-view radiograph at one-year follow-up showed K-wire backed out (**d**)

A free-hand TBW procedure was conducted in the same way as in the group that used 3D-printed, but without the use of a 3D model and a guide plate. The K-wires were inserted according to the surgeon's experience during the operation. A representative free-hand TBW case is shown in Fig. 2.

#### Postoperative treatment

Both groups received the same immediate postoperative care. None of the patients received additional external fixation. All the patients were allowed to elbow functional exercises after wound healing.

#### Outcome evaluation

The operative time and numbers of fluoroscopy were recorded. The K-wires position was assessed using the postoperative radiographs of the elbow. The success rate of the K-wires across the proximal fracture site of the olecranon and penetrated the distal anterior cortex was recorded. Follow-up was scheduled for postoperative 1, 3, 6, and 12 months. The functional scores of elbow and X-rays were assessed at each follow-up. Visual analog scale (VAS) of pain was checked. And the effect of clinical treatment was evaluated using the Mayo Elbow Performance Score (MEPS).

### Statistical analysis

The statistical analysis was conducted using the SPSS software, version 26 (IBM Corp. Armonk, NY, USA). Numbers and percentages were used to report descriptive statistics, and those for continuous variables were given as means ± SD. Categorical variables were analyzed using the Chi-square test, while continuous variables were analyzed using the Student’s *t*-test or the Mann–Whitney *U*-test. It was considered significant when the *P* value was less than 0.05 (two-sided).

## Results

### Baseline data

A total of 51 patients underwent TBW, 21 patients had individualized 3D-Printed template assisted surgery, and 30 patients had a free-hand TBW surgery. In the 3D-Printed group (12 males, 9 females; average age 45.29 ± 17.51 years), there were 15 cases with Mayo type 2A and 6 cases with Mayo type 2B. In the free-hand group (15 males, 15 females; average age 48.63 ± 19.13 years), there were 24 cases with Mayo type 2A and 6 cases with Mayo type 2B. As shown in Table 1, the baseline characteristics of participants were similar between both groups.

**Table 1:** Comparison of the basic data of patients in the two groups

	3D-Printed group (*n* = 21)	Free-hand group (*n* = 30)	*P* value
Age (years)	45.29 ± 17.51	48.63 ± 19.13	0.53
Gender (male/female)	12/9	15/15	0.62
Mayo type			0.48
2A	15	24	
2B	6	6	
Trauma			0.48
Traffic accident	15	24	
Fall	6	6	
Comorbidities			
Hypertension	14 (35.9%)	9 (29.0%)	0.83
Diabetes	5 (12.8%)	3 (9.6%)	0.62
Follow-up time (month)	14.33 ± 4.68	15.00 ± 7.64	0.72

### Clinical outcomes

There were no statistically significant differences between the two groups in operation time, VAS scores, and MEPS. There were fewer times of intraoperative fluoroscopy in the 3D-Printed group (1.43 ± 0.51) than that in the free-hand group (2.60 ± 1.00) with statistical significance (*P* < 0.05). The success rate of the K-wire across the proximal fracture site of the olecranon and penetrated the distal anterior cortex was 85.7% in the 3D-Printed group was significantly higher than the free-hand group (60%). At the date of the last follow-up, four patients suffer from pain and skin injury at the K-wires insertion site in the 3D-Printed group and 14 patients in the free-hand group, a significant difference between the two groups (*P* < 0.05). Clinical outcomes are summarized in Table 2.

**Table 2:** Comparison of clinical outcomes of patients in the two groups

	3D-Printed group (*n* = 21)	Free-hand group (*n* = 30)	*P* value
Operation time (minutes)	102.86 ± 14.96	107.33 ± 17.01	0.34
Fluoroscopy times	1.43 ± 0.51	2.60 ± 1.00	0.00
VAS scores	0.90 ± 1.18	0.93 ± 1.26	0.94
MEPS	92.76 ± 7.16	93.77 ± 6.69	0.61
Position of k-wire	18 (85.7%)	18 (60%)	0.04
Complications	4	14	0.04

*VAS* Visual Analog Scale; *MEPS* Mayo Elbow Performance Score

## Discussion

Olecranon fracture is one of the common fractures in patients with upper limb fractures, the most common cause occurs when a person falls from ground level height. The Mayo classification is the most widely used method for olecranon fracture. the most frequent type of fracture was Mayo type II injuries (82%) [9]. According to the presence of comminution, fracture types are subdivided into subtypes A and B. It is common for Mayo type II fractures to be displaced and therefore require surgical intervention. TBW is often used to treat simple olecranon fractures since it is known for its cost-effectiveness and biomechanical suitability [10]. The TBW technique requires two parallel K-wires (1.6 mm) to be placed antegrade across the fracture site. The distal end of the K-wire was allowed to be placed in the medullary cavity. However, one study [11] showed that the patients treated with intramedullary K-wires were found to have a higher rate of instability of K-wires than the patients treated with transcortical K-wires in TBW (78% vs. 36%). The instability of the K-wire will lead to pain and skin injury. The K-wires should penetrate the distal anterior cortex to reduce the risk of backing out proximally [11, 12].

In recent years, clinical applications of 3D-printed technology have increased as digital technology has advanced rapidly. The 3D-printed technology is often used in orthopedics for preoperative planning and designing guides for intraoperative navigation. The 3D models allow exquisite visualization of bone morphology and preoperative surgical simulation. Zhang et al. [13] reported that a 3D-Printed model to assist open fixation for calcaneal fractures effectively decreased complications, shortened operation times, and reduced hospitalization lengths. The greatest advantage of 3D-printed navigation templates in orthopedic surgery is the improved accuracy of internal fixation placement. Wu et al. [14] found that navigation templates assisted the C2 pedicle screw insertion resulting in a more accurate and safe procedure than traditional techniques. Similar results regarding the improvement of surgical accuracy by navigation templates have been reported in other studies [15, 16]. One other benefit of using a 3D printed model is that 3D printed model can be used as an auxiliary tool for preoperative communication with the patient.

With the use of 3D-printed technology, we fabricated an individualized navigation template in this study. Surgical simulations were used to determine the feasibility of the template. The success rate of the bicortical placement of k-wires and the effectiveness of this technique were evaluated and compared with traditional free-hand surgery. In our study, the success rate of the bicortical placement of k-wires was 85.7% in the 3D Printed group was significantly higher than the free-hand group (60%). Chalidis et al. [17] discovered that the accuracy of K-wires position with free-hand technology was 62.9%, the result of the study was similar to our study. A recent systematic review [18] found that the accuracy of pedicle screw placement can be improved using a 3D-printed template in cervical surgery. Li et al. [19] showed that the 3D-printed template improved osteotomy accuracy in cubitus varus deformity compared to traditional osteotomy. Similar to these studies, we found the 3D printed technique was an intuitive and efficient aid in TBW for treating olecranon fractures, which can improve the success rate of the free-hand bicortical placement of K-wires and reduce empirical errors.

Intramedullary K-wires were associated with a higher incidence of pain and skin injury in TBW for olecranon fracture. In our study, four patients suffer from pain and skin injury at the K-wires insertion site in the 3D-Printed group and 14 patients in the traditional group. The major causes of this local skin complication are the k-wire migration. According to Saeed et al. [20], intramedullary K-wire migration was more severe than that of transcortical K-wires (5.5 mm vs. 2.4 mm). Biomechanical studies [4, 21, 22] have confirmed that the insertion of K-wires into the anterior ulnar cortex has greater tensile strength and thus reduces proximal K-wire backout. This finding was supported by a previous study, by Rantalaiho et al. [23] demonstrated that K-wires inserted intramedullary had significantly more early complications than those transcortical K-wires.

In the present study, we found fewer times of intraoperative fluoroscopy in the 3D printed group than that in the free-hand group. The K-wires position was determined intraoperatively using C-arm radiography in TBW. When the X-ray showed that the distal end of the K-wire was in the ulnar bone marrow cavity, a second attempt was made to reinsert it. Re-adjust the position of the K-wire will increase the fluoroscopy times; however, to minimize the holding force and stability of the K-wire, repeated attempts should be abandoned. Kovalenko et al. [24] reported that the use of individual navigation templates for pedicle screw placement could significantly reduce the times of intraoperative fluoroscopy. In a study by Hu et al. [25], PVP with 3D-printed navigation templates could reduce fluoroscopy shot times and fluoroscopy dosage during surgery. This study demonstrated that a 3d printed model and navigation template could significantly reduce fluoroscopy times in TBW for olecranon fractures.

This study also has some limitations: First, this was a single-center study with a limited sample size. It might be more convincing to conclude a prospective study with large sample size. Second, the inaccurate placement of navigation templates may affect the accuracy of surgical procedures. Furthermore, 3D-printed and template production need to wait for 2 to 3 days, which will increase the length of hospital stay of patients. This technique is not suitable for emergency surgery patients.

In conclusion, the individualized 3D-printed navigation template-assisted TBW demonstrated good accuracy and resulted in reduced times of intraoperative fluoroscopy and complication compared to the free-hand TBW for olecranon fractures.

## Data Availability

The datasets generated and/or analysed during the current study are not publicly available due data that are the private property of the authors prior to publication of the study may be compromised but are available from the corresponding author on reasonable request.
